# Comprehensive review on neprilysin (NEP) inhibitors: design, structure-activity relationships, and clinical applications

**DOI:** 10.3389/fphar.2024.1501407

**Published:** 2024-12-23

**Authors:** Xinyue Zhang, Chun Hu, Erkang Tian, Yanxin Shen, Wei Liu, Juan Li

**Affiliations:** ^1^ Department of Stomatology, Chengdu Fifth People’s Hospital/The Second Clinical Medical College, Chengdu University of TCM, Chengdu, Sichuan, China; ^2^ Department of Orthodontics, State Key Laboratory of Oral Diseases, West China School of Stomatology, Sichuan University, Chengdu, Sichuan, China

**Keywords:** neprilysin, NEP inhibitor, structure-activity relationship, drug design, clinical

## Abstract

Neprilysin (NEP), a zinc-dependent membrane-bound metallopeptidase, regulates various bioactive peptides, particularly in kidneys, vascular endothelium, and the central nervous system. NEP’s involvement in metabolizing natriuretic peptides, insulin, and enkephalins makes it a promising target for treating cardiovascular and Alzheimer’s diseases. Several NEP inhibitors, such as sacubitril and omapatrilat, have been approved for clinical use, which inhibit NEP activity to prolong the bioactivity of beneficial peptides, thereby exerting therapeutic effects. However, despite the broad clinical application prospects of NEP inhibitors, they still have specific adverse reactions and side effects, such as hypotension, renal impairment, and a potentially increased risk of Alzheimer’s disease. This manuscript comprehensively reviews the progress on single-target and dual-target NEP inhibitors. Dual-target inhibitors often combine with other therapeutic targets, such as angiotensin receptors, to enhance therapeutic effects and reduce adverse reactions. The article also emphasizes these inhibitors' design strategies, structure-activity relationships (SAR), safety, and clinical performance.

## 1 Introduction

Neprilysin (EC 3.4.24.11, neutral endopeptidase, NEP), is widely found in tissues such as the central nervous system, kidneys, and vascular endothelium (Turner et al., 2001). NEP can degrade a diverse range of peptide substrates such as insulin, enkephalins, substance P, endothelin-1 (ET-1), and amyloid-β (Kerr and Kenny, 1974a; Skidgel et al., 1984; Howell et al., 1995). It cleaves these substrates at multiple sites, favoring the amino side of hydrophobic residues (Southwood et al., 1975). The extensive presence of NEP, coupled with its capacity to interact with various substrates, highlights its critical involvement in the physiological processes of the cardiovascular, renal, gastrointestinal, and neurological systems.

Beyond its broad distribution, NEP is crucial for processing and breaking down vasoactive peptides involved in diuresis and natriuresis. Essential peptides include natriuretic peptides (NP), angiotensin I (Ang I), angiotensin II (Ang II), adrenomedullin (ADM), bradykinin (BK), neurokinin A, neuropeptide Y, substance P, and ET-1 (Dorofeyeva, 1975; Stephenson and Kenny, 1987; Abassi et al., 1992; Jiang et al., 2004; Mangiafico et al., 2013).

Neprilysin (NEP) plays a crucial role in the regulation of cardiovascular and inflammatory processes by degrading various bioactive peptides, including angiotensin-(1-7) [Ang-(1-7)]. Ang-(1-7), which is converted from Ang II by angiotensin-converting enzyme 2 (ACE2), has vasodilatory and anti-inflammatory effects, opposing the vasoconstrictive and pro-inflammatory actions of Ang II. Inhibition of NEP can increase the levels of Ang-(1-7), thereby enhancing its protective effects on the cardiovascular system, such as lowering blood pressure, reducing cardiac remodeling, and suppressing inflammatory responses (Mukhomedzyanov et al., 2024). This mechanism is particularly important for the treatment of diseases like heart failure and hypertension (Medina and Arnold, 2019). Therefore, NEP inhibitors, by enhancing the activity of the ACE2/Ang-(1-7) axis, help to mitigate the impact of cardiovascular diseases and inflammatory events (Derkachev et al., 2024). NEP’s activity extends beyond the cardiovascular system, influencing neurological processes, pain, inflammation, mitosis, angiogenesis, and digestion (Jaffe et al., 2015; Riddell and Vader, 2017). Therefore, NEP has appeared as a potentially beneficial target for treating many diseases.

Neprilysin inhibitors (NEPI) can influence the metabolism of multiple peptides; previous studies have confirmed that inhibiting NEP can elevate the concentration of these peptides, improving the therapeutic effects for patients with chronic heart failure (Bayes-Genis et al., 2016). Moreover, NEPI is correlated with Alzheimer’s disease, cardiovascular diseases, arthritis, and other conditions. For example, inhibiting NEP activity can boost glucose-stimulated insulin secretion in isolated islet tissues from C57BL/6 mice (Esser et al., 2018) and promote skeletal growth through the CNP/NPR-B pathway (Hakata et al., 2024). In high-fat-fed NEP-deficient mice, improvements in insulin sensitivity, β-cell function, glucose tolerance, and β-cell proliferation have been observed (Willard et al., 2017; Parilla et al., 2018). Furthermore, Alzheimer’s disease (AD) is a heterogeneous neurodegenerative disorder marked by intracellular tau protein accumulation and extracellular β-amyloid (Aβ) deposition. Research indicates that NEP is one of the essential metalloproteases involved in the clearance of Aβ (Grimm et al., 2014), and its presynaptic neuronal localization aids in Aβ clearance. Thus, NEPIs may accelerate AD neuropathology by inhibiting the Aβ clearance pathway.

In recent years, efforts have been devoted to developing highly efficient and specific NEPIs, with some achieving promising results. Dual-target NEPIs, such as omapatrilat and fasidotril, have beneficial effects in treating cardiovascular diseases (C Marie et al., 1995; Sharma et al., 2020). However, the adverse reactions of NEPI also warrant attention. Some animal studies suggest potential negative impacts on cognitive function from NEPI, along with mild side effects such as vomiting, drowsiness, dizziness, and edema (Moreau et al., 2005). This paper reviews the progress in NEPI research, detailing the design strategies, structure-activity relationships (SAR), and bioactivity of single-target and dual-target NEPIs.

## 2 The structural and biological function of NEP and NEPI

### 2.1 The structure of NEP

NEP, a type II membrane-bound enzyme, comprises 749 amino acid residues and relies on zinc for its catalytic activity as an endopeptidase (Kerr and Kenny, 1974b; Skidgel et al., 1984; Howell et al., 1995). Its extracellular domain, part of the peptidase M13 family, features two α-helical structures forming a spherical, water-filled cleft that houses the catalytic site (Campbell, 2017). This domain resembles the bacterial protease thermolysin and contains a zinc atom essential for its catalytic activity, coordinated by histidine (His) and glutamate (Glu) residues. NEP also includes a transmembrane domain and a short intracellular domain. Located on the plasma membrane, NEP has three domains: a short intracellular domain (27 residues), a transmembrane domain (23 residues), and a larger extracellular catalytic domain (699 residues) (Moss et al., 2020). The extracellular region features a central hollow space that houses a key zinc-binding sequence, HEXXH, wherein a pair of histidine molecules anchor the zinc atom. At the same time, a glutamate residue is involved in the catalytic process (Moss et al., 2018).

**FIGURE 1:**
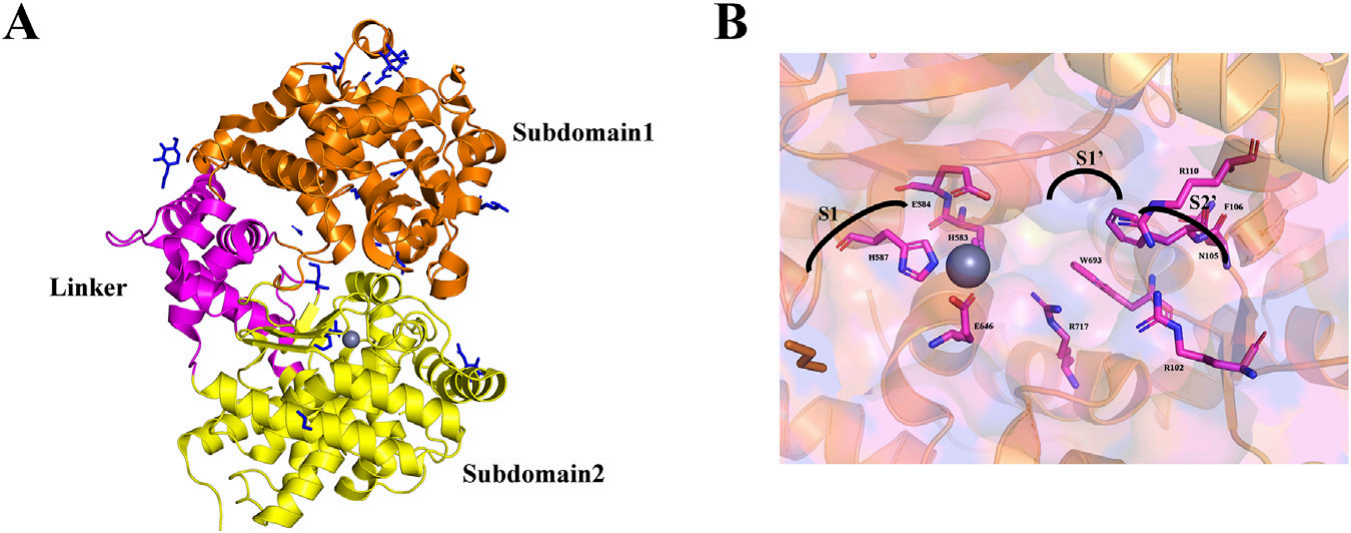
**(A)** The 3D ribbon diagram of the extracellular domain of NEP. The extracellular domain is further split into two subdomains colored orange for subdomain 1, yellow for subdomain 2, and pink for the linker region. **(B)** NEP active site binding pocket labeled with subsites and residues.

The extracellular region of NEP is ellipsoidal, with major and minor axes measuring 86 and 60 nm. It has a predominantly α-helical secondary structure, divided into two large subdomains connected by a smaller linker region comprising four α-helical segments (Figure 1A). Subdomain one contains the N-terminal residues, while subdomain two contains the C-terminal residues, creating a central cavity. Four N-linked glycosylation sites are at positions N144, N284, and N324 in subdomain two and N627 in subdomain 1, with a single N-acetylglucosamine residue modeled at each site (Oefner et al., 2000a).

The catalytic site within subdomain 1’s cavity surface centers around a zinc ion and the conserved HEXXH motif. The zinc ion is coordinated by residues H583 (2.02 Å), H587 (2.10 Å), and E646 (1.93 Å), with E584 contributing to the catalytic mechanism and completing the coordination sphere. This forms a binding pocket with sub-sites S1, S1', and S2'. Inhibitors targeting NEP reveal the characteristics of these sub-sites (Figure 1B). The S1 site has minimal impact on binding affinity and exhibits relaxed specificity (Oefner et al., 2000b). The S1' sub-site forms a hydrophobic pocket for large hydrophobic and aromatic side chains (Tiraboschi et al., 1999), explaining NEP’s preference for cleaving at hydrophobic residues. The S2' sub-site can accommodate bulky side chains and shows relaxed specificity (Dion et al., 1997).

### 2.2 The biological functions of NEP

**FIGURE 2:**
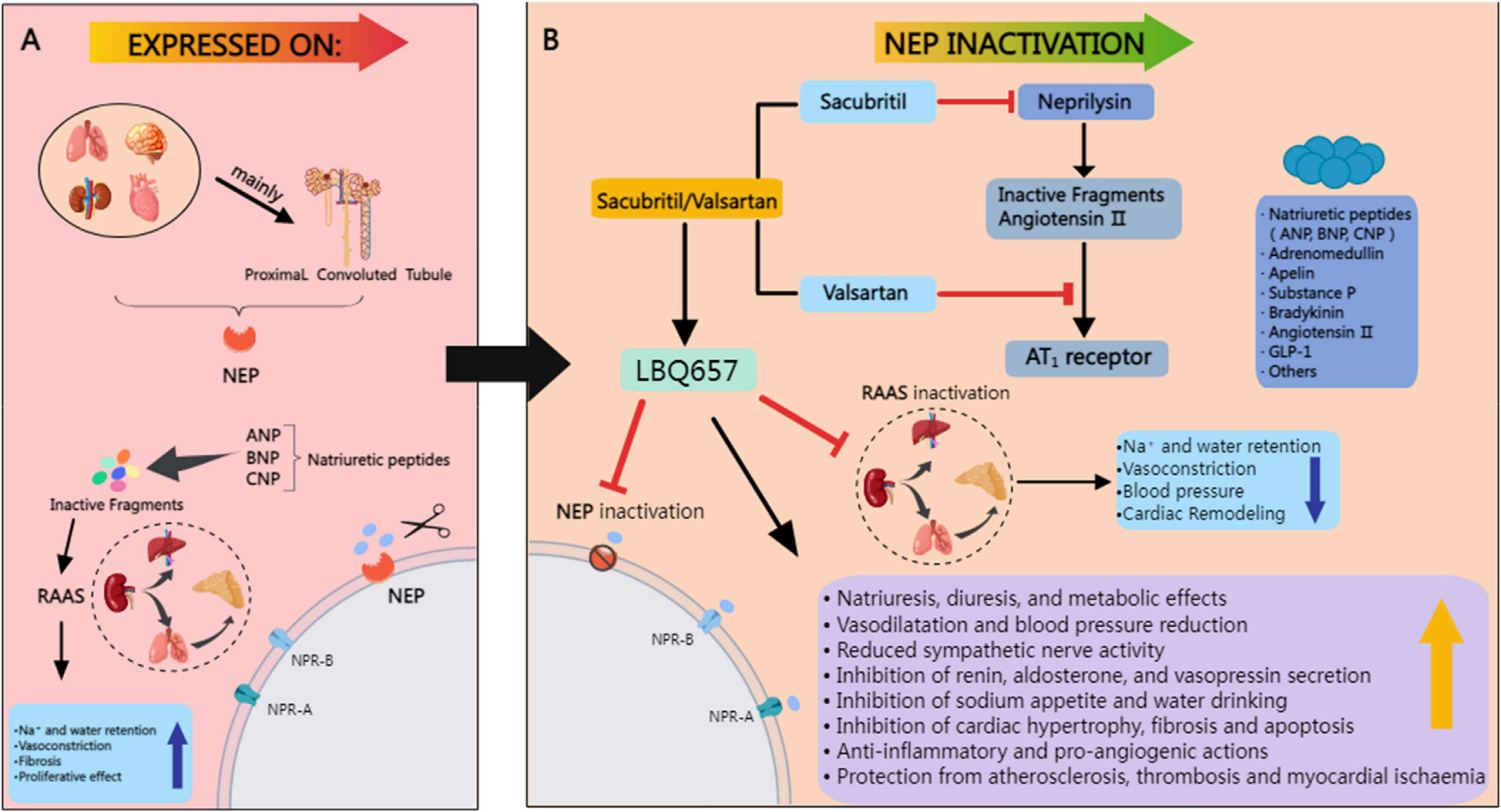
**(A)** NEP is abundantly present in various tissues, including the lungs, blood vessels, heart, brain, and kidneys, with the highest levels in the proximal tubules of the kidneys. It breaks down natriuretic peptides like ANP, BNP, and CNP, which inactivates the RAAS and promotes fluid retention. While beneficial for blood pressure regulation, this process raises the risk of cardiac metabolic problems due to its vasoconstrictive and antiproliferative effects. **(B)** It lowers arterial blood pressure by enhancing sodium excretion, promoting diuresis, and inducing blood vessel dilation, while also strengthening the anti-proliferative effects on heart remodeling, thus amplifying the hemodynamic effects of natriuretic peptides (NP). Red lines denote inhibitory actions. MedPeer. (2024). MedPeer Scientific Illustration System [Computer software]. Retrieved from http://www.medpeer.cn.

NEP belongs to the M13 peptidase family on chromosome 3q25.2 (Polna and Aleksandrowicz, 1975; Schiering et al., 2016). NEP has 749 amino acids and three main domains: a short amino-terminal cytoplasmic domain, a single transmembrane helical domain, and a carboxy-terminal extracellular domain containing a zinc-binding active site. These domains enable the enzyme’s catalytic activity when substrates attach to the extracellular domain (Emoto and Yanagisawa, 1995). NEP is an enzyme specializing in cleaving peptides with a molecular weight that generally does not exceed 3,000 Da. It does not act on larger proteins with a molecular weight well above this threshold. The limited access to the catalytic cleft determines the enzyme’s size-specific activity, which only allows substrates of a specific size to enter (Figure 2) (Oefner et al., 2000a). NEP catalyzes the hydrolysis of peptide bonds at the amino side of hydrophobic residues, with a particular affinity for phenylalanine or leucine at the P1 position. It cleaves the substrate at the carboxyl terminus, releasing dipeptides or tripeptides (Campbell, 2017). NEP catalyzes the hydrolysis of hydrophobic amino acids located at the N-terminus of peptides, showing a particular affinity for phenylalanine and leucine.

In addition to its broad distribution, neprilysin is instrumental in the metabolism and processing of various vasoactive peptides that are pivotal for diuresis and natriuresis. Key among these peptides are NP, Ang I, ADM, BK, neurokinin A, neuropeptide Y, substance P, and ET-1, all of which are subject to the enzyme’s catalytic action (Dorofeyeva, 1975; Stephenson and Kenny, 1987; Abassi et al., 1992; Jiang et al., 2004; Mangiafico et al., 2013). Neprilysin’s enzymatic function extends beyond the cardiovascular system; it also antagonizes a range of molecules integral to neurological functions, pain perception, inflammatory responses, cell division, blood vessel formation, and the digestive process (Jaffe et al., 2015; Riddell and Vader, 2017).

The blockade of two clearance mechanisms of NEP by ARNIs is a pivotal aspect of their therapeutic action. NEP, also known as neprilysin, is a membrane-bound metallopeptidase that plays a critical role in the degradation of various bioactive peptides, including atrial natriuretic peptide (ANP), brain natriuretic peptide (BNP), and substance P (Turner et al., 2001). The dual inhibition of NEP by ARNIs results in the blockade of two primary clearance pathways: the degradation of natriuretic peptides and the RAAS components (McMurray et al., 2014).

The first mechanism involves inhibiting natriuretic peptide degradation, leading to increased levels of these vasodilatory and antihypertrophic peptides, thereby enhancing their cardioprotective effects (Stuss, 2015). The second mechanism pertains to the blockade of the RAAS, where NEPI reduces the breakdown of angiotensin I and II, contributing to the attenuation of vasoconstriction and aldosterone release (Andersen et al., 1998).

### 2.3 The role of NEPI in diseases

NEPI affects the metabolism of various peptides, including amyloid peptides, corticotropin-releasing factor, luteinizing hormone-releasing hormone, oxytocin, and neurotensin (Bayes-Genis et al., 2016; Campbell, 2017). NEP plays a significant role in a variety of diseases affecting the natriuretic peptide system, the renin-angiotensin-aldosterone system (RAAS), and the kallikrein-kinin system (Domenig et al., 2016; Ponikowski et al., 2016). Previous studies have confirmed that inhibiting NEP can elevate these peptides, Improving chronic heart failure treatment outcomes (Campbell, 2017). NEPIs exert their effects by reducing the degradation of natriuretic peptides, thereby enhancing their bioactivity, which in turn promotes natriuresis, diuresis, vasodilation, inhibits cardiac hypertrophy and myocardial fibrosis, and delays the progression of heart failure (Pavo et al., 2020). Additionally, They may improve insulin and glucagon-like peptide-1 (GLP-1) levels by inhibiting the expression of dipeptidyl peptidase-4 (DPP4) (Pavo et al., 2020). Thus, by inhibiting NEP, the levels and functions of various bioactive peptides can be modulated, demonstrating potential biological functions and therapeutic potential in the treatment of a multitude of diseases.

#### 2.3.1 Diabetes and its complications

Studies have revealed that inhibiting NEP activity enhances insulin secretion stimulated by glucose in islet tissues extracted from C57BL/6 mice (Esser et al., 2018). Furthermore, *in vitro* experiments showed that NEP-deficient mouse islet tissues are protected from insulin secretion dysfunction (Zraika et al., 2013). *In vivo* studies have shown that NEP-deficient mice fed a high-fat diet demonstrate enhanced insulin sensitivity, improved β-cell function, better glucose tolerance, and increased β-cell proliferation (Willard et al., 2017; Parilla et al., 2018). Study reveals that under acute physiological conditions, selectively inhibiting intestinal neprilysin boosts insulin secretion in response to oral glucose, and this impact is mediated through the GLP-1 receptor, thereby establishing a novel role for intestinal neprilysin in the regulation of beta-cell function (Esser et al., 2023). NEPIs are also effective in the management of type 2 diabetes mellitus (T2DM) by increasing the circulating level of GLP-1, which is degraded by NEP (Esser and Zraika, 2019). However, the influence of other enzymes, such as DPP-4 inhibitors, on NEP substrates can diminish the efficacy of NEPI or adversely affect insulin sensitivity and β-cell function (Esser et al., 2022). As a result, the levels of NEP substrates may rise, suggesting that a combined NEPI treatment approach for type 2 diabetes could be more efficacious (AlAnazi et al., 2023).

Diabetic nephropathy (DN) is recognized as a prevalent cause of end-stage renal disease, driven significantly by chronic hyperglycemia activating the renin-angiotensin system (RAS). Therefore, blocking RAS can help delay the progression of DN (Gallagher and Suckling, 2016). The persistent hyperactivation of the RAS is implicated in the progression of diabetic nephropathy. Concurrently, the natriuretic peptide system (NPS) is a counterbalancing negative feedback mechanism (Goru et al., 2017; Malek and Gaikwad, 2017). NEPI can enhance the bioavailability of natriuretic peptides, which may contribute to mitigating chronic kidney disease (CKD) progression (Lai et al., 2023). Clinically, urinary NEP levels are increased in diabetic patients, and NEPI can delay DN progression. Furthermore, research indicates that treatment with angiotensin receptor-neprilysin inhibitors (ARNIs) can lead to improvements in renal function, reduce glomerular and tubulointerstitial fibrosis, and weaken inflammation, pro-fibrotic and apoptotic signaling, ultimately delaying DN onset (Davis et al., 2022).

Diabetic retinopathy (DR) stands as the leading cause of vision loss among individuals with diabetes. DR is recognized as a progressive neurovascular condition affecting the retina, with its severity and progression closely linked to the duration of diabetes. Medications such as angiotensin receptor blockers (ARBs) and ARNIs have demonstrated the ability to significantly attenuate NEP activity (Dahrouj et al., 2013). Inhibiting retinal NEP activity and increasing retinal natriuretic peptide levels can improve DR treatment outcomes, and ARNIs can improve neurovascular lesions in the retinas of diabetic rats (Prasad et al., 2016).

Recent advancements in the field of diabetic complications have shed light on the potential therapeutic roles of neprilysin inhibitors, both as monotherapy and in combination with other agents, in the management of diabetic nephropathy (DKD) and diabetic cardiomyopathy (DCM). In a study, the combination of SGLT2 inhibitors with neprilysin inhibitors demonstrated promising results in improving glycemic control and reducing inflammation and oxidative stress, which are key factors in the progression of DKD (Tarun et al., 2024). Furthermore, the synergistic effects of these drugs on lowering glomerular pressure and proteinuria offer a novel approach to DKD management.

Residing on the cell surface of the corneal epithelium, where it remains undisturbed, NEP plays a pivotal role in controlling the chromatic interactions between corneal nerve fibers and the epithelial cells themselves through the modulation of peptides involved in these communications. Studies have shown that NEPI combined with topical ascorbic acid, citric acid, antibiotics, or steroids helps improve epithelial replanting on the corneal surface (Genova et al., 2018). Although a previous study has documented the presence of NEP in the human corneal epithelium, the exact physiological functions of this enzyme in this context have yet to be fully elucidated (Hasby and Saad, 2013).

#### 2.3.2 Chronic kidney disease

In hypertensive animal models, long-term administration of omapatrilat has been observed to effectively halt the advancement of renal complications, including glomerulosclerosis, tubulointerstitial fibrosis, and overall kidney injury (Cao et al., 2001). In a 5/6 nephrectomy model, treatment with AVE 7688 has demonstrated significant efficacy in Mitigating proteinuria and ameliorating the severity of glomerulosclerosis and tubulointerstitial fibrosis (Benigni et al., 2004). AVE 7688 increased renal nitric oxide compounds, reduced endothelin-1 synthesis, decreased renal vascular contraction, and raised tubular ANP release (Benigni et al., 2004). In another 5/6 nephrectomy model, omapatrilat lowered systolic blood pressure and glomerular capillary pressure, reduced proteinuria, and mitigated glomerulosclerosis (Taal et al., 2001). Therefore, combined NEPI can improve CKD treatment outcomes.

#### 2.3.3 Colitis

Ulcerative Colitis is an idiopathic chronic inflammatory condition that targets the colon’s mucosal lining, leading to relapsing and remitting symptoms. The absence or inhibition of NEP can exacerbate *Clostridium difficile* toxin A-induced intestinal inflammation (Sargın et al., 2017). Additionally, recombinant NEP can prevent inflammation in NEP knockout mice. A significant deficiency in NEP activity could contribute to a pro-inflammatory environment within the colonic mucosa (Ter Beek et al., 2007). The inflammatory state is linked to NEP and substance P (SP) loss. The pro-inflammatory action of low SP levels may be related to bioactive fragments. In UC, the metabolism of peptides that act as pathogenic agents is likely impaired due to a substantial decrease in the activity of the principal enzyme that catalyzes their hydrolysis.

Additional investigative efforts are warranted to elucidate the underlying reasons for the observed decline in peptide levels. Vu et al.'s research confirms Vasoactive Intestinal Peptide (VIP) as an anti-inflammatory agent, showing reduced DSS-induced Colitis in both VIP knockout mice and wild-type mice treated with VIP antagonists (Vu et al., 2014), NEP Deficiency might dampen VIP anti-inflammatory-action-by-affecting production of its active VPAC1-binding fragments (Pavlovic et al., 2011). In the colonic tissues of UC patients, the expression of SP and VIP significantly decreases, and NEP expression is markedly reduced.

#### 2.3.4 Alzheimer’s disease

Alzheimer’s disease manifests as a neurodegenerative condition with hallmark pathologies: the intracellular presence of phosphorylated tau and the extracellular presence of β-amyloid plaques (Nalivaeva et al., 2020; Yiannopoulou and Papageorgiou, 2020). NEP is associated with the degradation and clearance of Aβ. NEP, a crucial metalloprotease, plays a significant role in the degradation and clearance of Aβ peptides (Grimm et al., 2014), and its presynaptic neuronal localization aids in Aβ removal (Iwata et al., 2004). In late-onset AD patients, there is a selective reduction in NEP mRNA expression and protein levels in brain areas. The decline in NEP levels and activity with age may contribute to late-onset Alzheimer’s disease in both humans and rodents (Iijima-Ando et al., 2008). Preclinical research suggests that the administration of NEPI may trigger Alzheimer ‘s-like pathologies in animal models (Liu et al., 2010). In mice (Poirier et al., 2006), NEPI exacerbates AD progression. A deficiency in NEP leads to impaired degradation of exogenous Aβ and disruptions in the metabolic regulation of endogenous Aβ levels. In the brains of NEP-deficient mice, Aβ levels are highest in the hippocampus, followed by the cortex. The thalamus/striatum, and lowest in the cerebellum, mirroring the pattern of vulnerability to Aβ deposition observed in the brains of AD patients (Liu et al., 2009; Liu et al., 2010; Pennant et al., 2014). Observations indicate (Marr and Spencer, 2010) that even a slight reduction in NEP activity can lead to AD development by facilitating the buildup of Aβ peptides. Studies (Ali et al., 2024) have suggested that NEPI might offer protective effects against the development of AD by increasing the levels of GLP-1, neuropeptide Y (NPY), and substance P. However, NEPI might contribute to AD development by increasing levels of BK and NPs (Bavishi et al., 2015). The contradictory findings on NEPIs' impact on AD progression are yet to be conclusively established in clinical settings.

#### 2.3.5 Heart failure (HF)

HF represents the end stage of various cardiac diseases. Inhibiting the excessive activation of the neuroendocrine system is a crucial measure in the treatment of HF. However, traditional drug treatments based on angiotensin-converting enzyme inhibitors (ACEI) and angiotensin II receptor blockers (ARB) have been found to exhibit an “upper limit effect,” where the effectiveness of the drugs diminishes or is even lacking in some long-term users, leading to reduced clinical outcomes (Arundel et al., 2020). NEP plays a significant role in the pathogenesis and development of HF by affecting the natriuretic peptide system, the RAAS, and the kallikrein-kinin system (Domenig et al., 2016; Ponikowski et al., 2016). In 2006, the first ARNI—sacubitril/valsartan was introduced. Extensive clinical trials have confirmed that ARNI offers significant benefits in the treatment of heart failure with reduced ejection fraction (HFrEF), new-onset HF, and acute decompensated HF, as well as in improving prognosis (Seferovic et al., 2019). Furthermore, the apelin/label system plays a crucial role in the cardiovascular system including the regulation of vascular tension, blood pressure, and fluid homeostasis (Kuba et al., 2019). Recent studies have indicated that neprilysin can cleave and inactivate apelin peptides, suggesting that neprilysin may indirectly modulate the function of the apelin/label system by affecting the stability and activity of apelin peptides (Chapman et al., 2021).

## 3 NEP inhibitors

### 3.1 Single-target NEPIs

**FIGURE 3:**
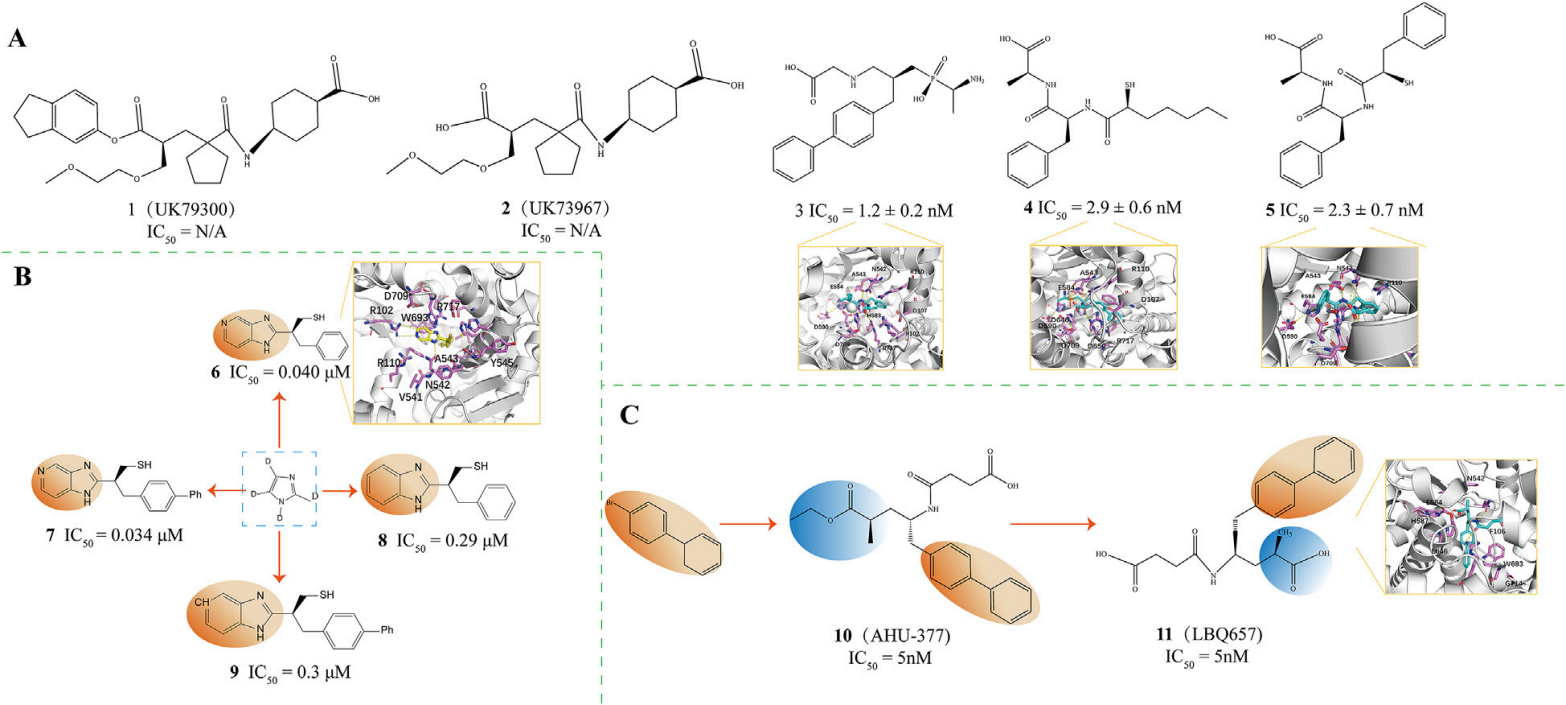
**(A)** NEP inhibitors. X-ray structure of NEP in complex with compound **3**(PDB ID: 1R1H), compound **4**(PDB ID: 1R1I), compound 5(PDB ID: 1R1J) (O’Connell et al., 1992; Oefner et al., 2004). **(B)** NEP inhibitors. X-ray structure of NEP in complex with compound 6(PDB ID: 1Y8J) (Sahli et al., 2005). **(C)** Structural Optimization of Compounds **10**,**11**. Crystal structure of compound **11**(PDB ID: 5JMY) (Flick et al., 2017; He et al., 2023).

Candoxatril (Compound **1**, UK79300, Figure 3A), developed by Pfizer, is a prodrug of Candoxatrilat (compound **2**, UK73967, Figure 3A) when taken orally. Candoxatril is a novel orally active neutral endopeptidase inhibitor involving the peptide hormone atrial natriuretic factor (ANF) metabolic inactivation (O’Connell et al., 1992). During development, the introduction of chirality was most effectively achieved using a BINAP-based catalyst for asymmetric hydrogenation (Ager and De Vries, 2012). Candoxatril, a hormone composed of 28 amino acids, contributes to the maintenance of sodium and water balance within the body (O’Connell et al., 1992), and is well-tolerated in humans (Yandle et al., 1986; Ignatovich, 1975; Mukoyama et al., 1991; Cheung et al., 1994). Exogenous administration of atrial natriuretic peptide (ANP) results in natriuresis (Epstein et al., 1998; Gunning et al., 1992), vasodilation (Fujita et al., 1987; Edwards et al., 1988) and reduced plasma concentrations of renin and aldosterone (Steele et al., 1997), thereby alleviating symptoms of heart failure.

Candoxatrilat is a neutral endopeptidase inhibitor that reduces ANP degradation in mild heart failure patients (Wilkins et al., 1992). Candoxatrilat and SC 46542 (an antagonist of ANP clearance receptors) inhibit two major mechanisms for clearing ANP - the enzyme E−24.11 and C-ANP receptors respectively. When administered alone at “physiological” plasma ANP levels, candoxatrilat does not significantly affect sodium excretion (Wilkins et al., 1992). The simultaneous blockade of two clearance mechanisms, namely, the enzyme E-24.11 and the C-ANP receptors, has yielded a more pronounced elevation of plasma ANP levels and a more robust natriuretic effect than inhibiting a single pathway (Polna and Aleksandrowicz, 1975; Rajcáni et al., 1975). Likewise, the clearance systems become more overwhelmed when plasma ANP levels are increased—whether through peptide infusion or under pathological states like heart failure. The capacity for one clearance mechanism to make up for the suppression of another is restricted, thereby highlighting the natriuretic capacity of candoxatrilat (Wilkins et al., 1992). Comparisons of the renal effects of candoxatrilat with low-dose and high-dose ANP infusions indicate different mechanisms for increasing sodium excretion, suggesting that the natriuretic response to ANP results from actions on both glomerular and tubular levels (Cogan, 1990). Researchers observed that the addition of candoxatrilat or SC 46542 to ANP at a dosage of 100 ng·kg^-^1·min^-^1, as well as the combined administration of candoxatrilat and SC 46542, led to an increase in urinary sodium and cyclic GMP excretion to levels similar to or higher than those achieved with ANP at 300 ng kg^-1^ min^-1^, however, these compounds were unable to replicate all the effects of high-dose ANP infusion (Chien et al., 1987; Trippodo and Barbee, 1987). These data suggest that candoxatrilat selectively amplifies the renal impact of ANP in conscious, normotensive rats, surpassing the effects achieved with medicinal dosages of ANP (Wilkins et al., 1992).

Oefner and colleagues investigated the interactions of stable inhibitors with NEP Through X-ray crystallography to understand enzyme inhibition patterns better. Among these, compound **3** (IC_50_ = 1.2 ± 0.2 nM, PDB ID = 1R1H, Figure 3A) was found to bind NEP with a zinc ion in an almost tetrahedral coordination geometry. The single O atom of the phosphonate group forms a tetrahedral bond with the zinc ion, maintaining a bond length of 1.94 Å (Oefner et al., 2004). Among these, compound **3** was found to bind NEP with a zinc ion in an almost tetrahedral coordination geometry. The single O atom of the phosphonate group forms a tetrahedral bond with the zinc ion, maintaining a bond length of 1.94 Å(Pauptit et al., 1988). The free amino terminus of this inhibitor contributes to the stabilization of the complex by forming a hydrogen bond with the carbonyl oxygen atom of the Ala543 backbone (Oefner et al., 2004). Introducing the free amino group into compound **3** yielded compound **4** (IC_50_ = 2.9 ± 0.6 nM, PDB ID = 1R1I, Figure 3A) and compound **5** (IC_50_ = 2.3 ± 0.7 nM, PDB ID = 1R1J, Figure 3A) (Chen et al., 2000). The rearrangement structures of NEP binary complexes with compounds **4** and **5** indicate that both interact with the Zn atom through bidentate coordination (Oefner et al., 2004). Compounds 8 and 9 display identical C-terminal extensions, which engage in similar molecular engagements with the enzyme, underscoring the supportive role of the S1 subsite in stabilization (Oefner et al., 2004). Overall, for all three compounds, the L-alanine residue at the carboxyl terminus is uniformly recognized by the amino acids within the S2' subsite, particularly reaching out to interact with the side chains of Arg102, Asp107, and Arg110 (Oefner et al., 2004).

Sahli and colleagues have developed a class of non-peptidic inhibitors that target the zinc-dependent metalloprotease neprilysin, using aromatic heterocycles as the core structure. These inhibitors demonstrate nanomolar inhibitory potency, with IC_50_ values in the nanomolar activity range (0.034 ± 0.30 mm) (Sahli et al., 2005). They investigated a pair of distinct synthetic pathways, ultimately identifying a superior approach distinguished by incorporating a thiol group via a Mitsunobu reaction as the culminating step in a complex sequence of reactions, which coordinates with the ZnII ion of the metalloprotease. This approach led to significantly improved binding affinity of the novel inhibitors for neprilysin (Sahli et al., 2005). Notably, inhibitors that have an imidazo [4,5-c]pyridine core, such as compound **6** (IC_50_ = 0.040 μM, PDB ID: 1Y8J, Figure 3B) and compound **7** (IC_50_ = 0.034 μM, Figure 3B), as well as those with a benzimidazole core, such as compounds **8** (IC_50_ = 0.29 μM, Figure 3B) and **9** (IC_50_ = 0.3 μM, Figure 3B), have shown promising activity (Sahli et al., 2005). Compound **6** demonstrated a high affinity for neprilysin, characterized by forming hydrogen bonds with amino acid residues Asn542 and Arg717 and beneficial π-π stacking interactions with the imidazole ring of His711 (Sahli et al., 2005).

Sacubitril (Compound **10**, AHU-377, IC_50_ = 5 nM, Figure 3C) is a medication developed by Novartis, which functions as a combination of a neprilysin inhibitor and an ARB (Flick et al., 2017; He et al., 2023; Li et al., 2023; Smits et al., 2024). Sacubitril targets neprilysin, an enzyme degrading the plasma ANF. Sacubitril maintains endogenous ANF levels by inhibiting neprilysin, protecting the cardiac neuroendocrine system, which benefits heart failure patients. In animal models, sacubitril has increased sodium excretion in urine, indicating its diuretic effect (He et al., 2023).

LBQ657 (Compound **11**, IC_50_ = 5 nM, PDB ID = 5JMY, Figure 3C) is an active neutral endopeptidase inhibitor. It is an active metabolite of the prodrug AHU-377 formed *in vivo* through esterase-mediated demethylation (Ksander et al., 1995). The LBQ657 molecule contains two chiral centers, the precise configurations enabling the most favorable interactions with the active site of neprilysin (Schiering et al., 2016). The carbonyl and amine Groups within the amide backbone of LBQ657 engage in hydrogen bonding with the asparagine side chains at position 542 and arginine at position 717 (Schiering et al., 2016). LBQ657 features an amide-NH linkage to the chiral carbon atom, representing a structural deviation from prior inhibitors that usually connect through the amide’s carbonyl oxygen. Despite this difference, the nitrogen and oxygen atoms in LBQ657 retain similar spatial orientations and interaction patterns within the complex as those observed in earlier compounds (Oefner et al., 2000b; Oefner et al., 2004; Sahli et al., 2005). The succinate in P2' has been optimized to place the carboxyl group in an ideal position to interact with Arg 102 and Arg 110, which is unique to LBQ657 (Ksander et al., 1995). By inhibiting NEP, LBQ657 increases the levels of certain peptide hormones (such as ANP and BNP) with vasodilatory and natriuretic effects in the body. These peptides activate guanylate cyclase through their receptors, increasing intracellular cyclic GMP levels, thereby exerting their physiological effects, including vasodilation, promoting urine excretion, and suppressing the RAAS (He et al., 2023).

### 3.2 Dual-target inhibitors

#### 3.2.1 NEP and ACE dual-target inhibitors

**FIGURE 4:**
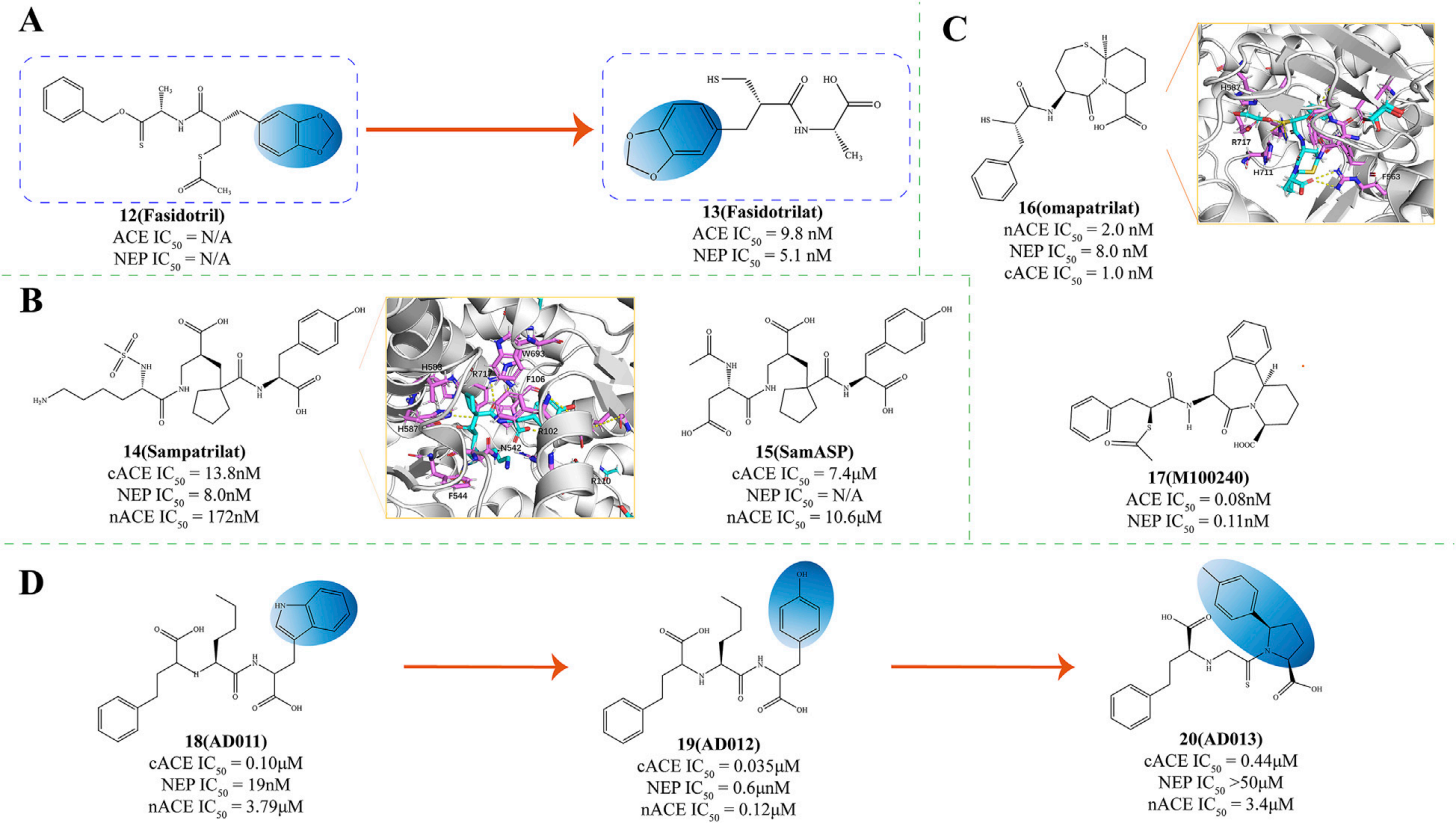
**(A)** Structural Optimization of Compounds **12**,**13** (C Marie et al., 1995). **(B)** Structures of NEP and ACE dual inhibitor (Wallis et al., 1998; Venn et al., 1998b; Venn et al., 1998a; Sharma et al., 2020). **(C)** Structures of NEP and ACE dual inhibitor. X-ray structure of NEP in complex with compound **16** (PDB ID: 6SUK) and compound **17** M100240 (Cozier et al., 2018; Rousso et al., 1999). **(D)** The structural optimization of ACE/NEP dual inhibitor (Oefner et al., 2000b; Arendse et al., 2022; Oefner et al., 2004; Sharma et al., 2020).

Fasidotril (Compound **12**, Figure 4A) is a diester prodrug developed by Bioproject. Its active metabolite, Fasidotrilat (Compound **13**, Figure 4A), effectively inhibits angiotensin I-converting enzyme (ACE IC_50_ = 9.8 nM) and neutral endopeptidase (NEP IC_50_ = 5.1 nM) (C Marie et al., 1995). In heart failure models, long-term treatment with fasidotril improves myocardial hypertrophy and increases survival rates without lowering blood pressure (C Marie et al., 1995). Studies have shown that in clinical studies, fasidotril strongly affects renin-dependent and volume-dependent hypertension models and can sustainably reduce blood pressure (C Marie et al., 1995). Fasidotril inhibits NEP, which reduces the degradation of ANP and BNP, enhancing their natriuretic and vasodilatory actions, thereby helping to alleviate the symptoms of heart failure (Pathadka et al., 2021). Additionally, by blocking ACE, fasidotril decreases the production of Ang II, mitigating its vasoconstrictive and pro-inflammatory actions, and offering therapeutic benefits for hypertension and myocardial infarction (Tsutsui et al., 2021). This makes fasidotril a promising candidate for treating myocardial infarction and congestive heart failure.

Sampatrilat (Compound **14**, UK 81252, Figure 4B) functions as a dual-action inhibitor targeting both ACE and NEP and it holds promise for therapeutic use in managing conditions such as hypertension and congestive heart failure (Wallis et al., 1998; Venn et al., 1998b; Venn et al., 1998a). Given its dual action, sampatrilat may offer more excellent benefits in treating chronic heart failure than traditional ACE inhibitors (Norton et al., 1999).

SamASP (Compound **15**, Figure 4B) interacts with NEP akin to sampatrilat, securing its position in the S2' and S1' subsites, the region surrounding the zinc ion, and extending its reach into the Non-prime subsites (Sharma et al., 2020). In the non-prime region, the secondary amide and aspartic acid-like side chains on the C5 position of SamASP offer a fitting interaction that aligns well with the enzyme’s binding requirements, with the carboxyl group of the tartaric ester located in the densest electron density regions. The secondary amide’s strategic placement enables its C1 and C2 atoms to engage in hydrophobic interactions with the Val-710 residue (Sharma et al., 2020).

Omapatrilat (Compound **16**, Vanlev, NEP IC_50_ = 8.0 nM, PDB ID: 6SUK, Figure 4C) is an investigative pharmaceutical that functions as an inhibitor for both NEP and ACE (Sharma et al., 2020). In clinical studies, omapatrilat has been extensively investigated as a dual ACEI/NEPI (Cozier et al., 2018; Robl et al., 1997), and has proven significant efficacy in lowering blood pressure among individuals with hypertension (Azizi et al., 2000; Kostis et al., 2004). However, compared to placebo, omapatrilat is associated with a notably higher occurrence of side effects such as coughing, skin flushing, short-term facial redness, gastrointestinal disturbances, and potentially life-threatening adverse reactions such as angioedema (Arendse et al., 2019). These adverse safety profiles have hindered the advancement of this promising vasopeptidase inhibitor.

Omapatrilat was crafted to mimic a tripeptide, allowing it to engage with the S1, S1', and S2' subsites of the target metalloproteases, a design intended to enhance its binding specificity and efficacy (Sharma et al., 2020). The primary binding occurs in the NEP complex structure at the S1' and S2' subsites. At the same time, a segment of the bicyclic group protrudes into the S3' region, facilitating a more extensive interaction with the enzyme’s binding pockets (Sharma et al., 2020). The thiol group of omapatrilat forms a coordination complex with the zinc ion and two water molecules, facilitating interactions with His711 and the backbone of Ala543 (Sharma et al., 2020). The phenyl moiety of omapatrilat delves into the S1' pocket, creating many hydrophobic contacts with Phe106, Phe563, Val580, and Trp693. Moreover, its Cα-analogous atom also participates in hydrophobic interactions with His583, thereby enhancing the compound’s binding to the NEP enzyme (Sharma et al., 2020). The P1′ carbonyl of omapatrilat has hydrophobic interactions with His-711 at C11 and bidentate interactions with Arg-717 at O4 (Sharma et al., 2020).

M100240 (Compound **17**, Figure 4C), a sulfur ester of MDL 100173, is a dual inhibitor targeting both angiotensin I-converting enzyme (ACE IC_50_ = 0.08 nmol/L) and neutral endopeptidase (NEP IC_50_ = 0.11 nmol/L) (Rousso et al., 1999). M100240 has been demonstrated in clinical research to decrease ACE activity and the concentration of angiotensin II. Simultaneously, it raises plasma renin levels and potentiates the beneficial actions of atrial natriuretic peptides (Rousso et al., 1999). This could offer distinctive therapeutic advantages in conditions marked by heightened vascular volume or sodium surplus and increased venous pressure (Schiering et al., 2016). Studies further suggest that M100240 exhibits safety and good tolerability in individuals, whether in a fed or fasting state (Schiering et al., 2016).

Lauren B. and the research team created three dipeptides with a carboxyl-3-phenyl propyl group at the terminal end and N-terminal nitrogen inspired by the structure of LisW—an ACE inhibitor. This synthesis was conducted to investigate the structural prerequisites for achieving dual inhibition of both ACE and NEP (Arendse et al., 2022). Removal of the P1′ amine from LisW to create AD 011 (Compound **18**, Figure 4D) improved NEPI potency but showed poorer NEP affinity compared to ACE affinity (Oefner et al., 2000b; Arendse et al., 2022). Substituting the P2′ tryptophan residue in AD 011 with tyrosine to produce AD 012 (Compound **19**, Figure 4D) further enhanced binding to NEP (Oefner et al., 2004; Arendse et al., 2022). In contrast to the earlier reported 2-mercapto-3-phenylpropionyl derivatives that possessed identical P1′ and P2′ groups, compound AD 013 (Compound **20**, Figure 4D) exhibited a diminished binding affinity for NEP and the catalytic domain of cACE (Sharma et al., 2020). For inhibitors lacking suitable P1′ side chains for NEP S1′ subsite binding, those with a 2-mercapto-3-phenylpropionyl N-terminal may adopt a more selective binding orientation in NEP (Arendse et al., 2022).

#### 3.2.2 NEP and ECE dual target inhibitors

**FIGURE 5:**
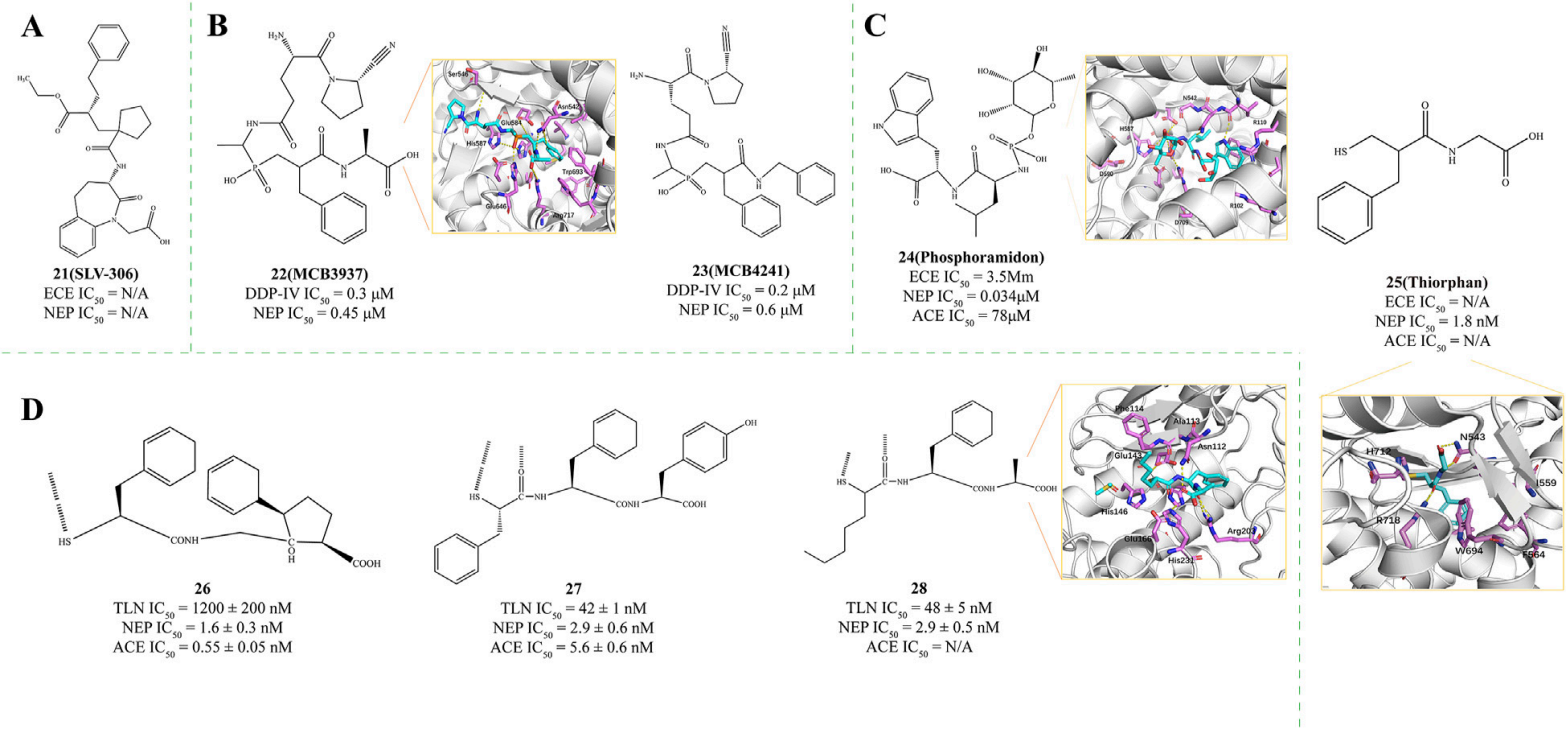
**(A)** Structures of NEP and ECE dual inhibitor (Seed et al., 2012). **(B)** Structures of NEP and DDP-IV dual inhibitor. X-ray structure of NEP in complex with compound **23** (PDB ID: 2QPJ) (Oefner et al., 2004). **(C)** Structures of NEP, ECE, and ACE Multi-Target Inhibitor. X-ray structure of NEP in complex with compound **24** (PDB ID: 1DMT) (Oefner et al., 2000b; Roques et al., 1980; Lambert et al., 1993; Labiuk et al., 2019). **(D)** Structures of NEP, TLN, and ACE Multi-Target Inhibitor. X-ray structure of NEP in complex with compound **28** (PDB ID: 1QF1) (Gaucher et al., 1999).

Daglutril (Compound **21**, SLV-306, Figure 5A) represents an innovative dual-action inhibitor targeting both NEP and ECE (Seed et al., 2012). The fundamental principle of this drug is to inhibit both neprilysin and endothelin-converting enzymes (Seed et al., 2012). The dual-action inhibitor’s benefit is bypassing the compensatory actions that occur with single enzyme inhibition, potentially offering better efficacy by blocking both enzymes simultaneously (Ferro et al., 1998). Following oral intake, Daglutril is metabolized into its active form (Dickstein et al., 2004), which then inhibits the systemic transformation of Big-ET1 and boosts circulating levels of ANP (Seed et al., 2012). In a compact, randomized, crossover, double-masked, placebo-controlled study, Daglutril was found to lower blood pressure in individuals with type 2 diabetic nephropathy throughout the 8-day treatment period, yet it did not demonstrate a reduction in albuminuria (Parvanova et al., 2013).

#### 3.2.3 NEP and DPP-IV dual target inhibitors

Dipeptidyl peptidase IV (DPP-IV) is a versatile type II transmembrane serine protease that functions as a glycoprotein. It comprises 766 amino acids: 6 amino acids are located within the cytoplasm, 22 amino acids comprise the portion that spans the plasma membrane, and the remaining 738 amino acids constitute the extracellular domain (Misumi et al., 1992). Oefner et al. proposed that DPP-IV inhibitors could be connected to NEPIs through their terminal unreacted substituents, residues, or C-terminal portions (Oefner et al., 2004). Following this design strategy, they synthesized NEP/DPP-IV dual inhibitors MCB 3937 (Compound **22**, DPP-IV IC_50_ = 0.3 μM, NEP IC_50_ = 0.45 μM, PDB ID: 2QPJ, Figure 5B) and MCB 4241 (Compound **23**, DPP-IV IC_50_ = 0.2 μM, NEP IC_50_ = 0.6 μM, Figure 5B) (Oefner et al., 2004). The benzyl group of the Inhibitors engages with the S1′ subsite of the enzyme, enhancing the binding affinity for NEP. This interaction is mainly facilitated by the aromatic or bulky hydrophobic residues present in the subsite (Oefner et al., 2004). The concept of dual NEP/DPP-IV inhibition can, in theory, be extended to a range of established enzyme inhibitor classes to potentially enhance their inhibitory efficacy.

#### 3.2.4 Angiotensin receptor neprilysin inhibitors

ARNIs are a novel class of cardiovascular drugs that have emerged as a significant advancement in the treatment of heart failure and hypertension. ARNIs exert their effects by inhibiting the angiotensin receptor and neprilysin, a neutral endopeptidase that degrades various vasoactive peptides, including natriuretic peptides (Yamamoto and Rakugi, 2021).

The first-in-class ARNI, sacubitril/valsartan, has demonstrated superior cardioprotective effects compared to traditional renin-angiotensin system inhibitors (RAS-Is) in large clinical trials such as the PARADIGM-HF trial (Tsukamoto et al., 2023). This drug combines the neprilysin inhibition of sacubitril with the angiotensin receptor blockade of valsartan, enhancing the levels of beneficial peptides like natriuretic peptides while blocking the effects of angiotensin II (Kario and Williams, 2022).

ARNIs are effective in reducing cardiovascular mortality and heart failure hospitalizations by 20% in ambulatory patients with heart failure and reduced ejection fraction, as reported in the PARADIGM-HF trial (Mann et al., 2022). They have also been suggested to provide renoprotective effects beyond those of RAS-Is in patients with heart failure (Tsukamoto et al., 2023).

Furthermore, ARNIs have been studied for their efficacy in blood pressure management. Accumulating evidence suggests that sacubitril/valsartan is superior to conventional RAS Blockers in lowering blood pressure in patients with hypertension (Bozkurt et al., 2023). This class of drugs is effective in improving renal outcomes and has a lower risk of renal dysfunction and a higher estimated glomerular filtration rate (eGFR) compared to RAS-Is, without an increased risk of hyperkalemia (Chopra et al., 2023).

### 3.3 Multi-target inhibitors

#### 3.3.1 NEP, ECE, and ACE multi-target inhibitors

Phosphoramidon (Compound **24**, Figure 5C) is a microbial metabolite and a specific metalloprotease thermolysin inhibitor. Phosphoramidon inhibits neprilysin (NEP, IC_50_ = 0.034 μM, PDB ID: 1DMT), as well as an endothelin-converting enzyme (ECE, IC_50_ = 3.5 μM) and angiotensin-converting enzyme (ACE, IC_50_ = 78 μM) (Oefner et al., 2000b). The rhamnose group at the P1 position of the inhibitor is mainly in contact with the solvent, allowing the majority of the phosphoramidon surface to engage with the enzyme, thereby providing a secondary stabilization effect on the S1 subsite (Gaucher et al., 1999; Ksander et al., 1997b). The NEP’s1 subsite creates a substantial hydrophobic pocket that selectively binds with aromatic or bulky hydrophobic moieties (Tiraboschi et al., 1999). The S2′ subsite, notably spacious, reaches into the solvent environment through the junctional helix A3, approaching the side chains of residues R102, D107, and R110. This observation verifies that the S2′ subsite of NEP exhibits lower specificity and can accommodate larger side chains. These polar residues are not involved in the binding process with the inhibitor (Lambert et al., 1993).

As a NEPI, Racecadotril can rapidly metabolize into the effective Thiorphan (compound **25**, IC_50_ = 1.8 nM, PDB ID = 5V48, Patent: US20110319656, Figure 5C) in the body (Roques et al., 1980; Lambert et al., 1993; Labiuk et al., 2019; Schwartz, 2000; Eberlin et al., 2012). In contrast, its inhibitory effects on ACE and Endothelin-Converting Enzyme 1 (ECE1) are significantly weaker, with Ki values exceeding 0.1 and 10 μM, respectively. Thiorphan exerts its inhibitory effect on NEP by binding to the zinc ion at the enzyme’s active site via its thiol group, a critical interaction for its activity (Labiuk et al., 2019). This coordination interferes with the catalytic mechanism of NEP, thereby inhibiting its peptidase activity (Labiuk et al., 2019). Additionally, the phenyl ring structure of Thiorphan penetrates the central cavity of NEP and interacts hydrophobically with surrounding residues such as Phe107, Ile559, Phe564, Val581, and Trp694. These hydrophobic interactions are essential for Thiorphan’s stable binding in the NEP active site, contributing to its inhibitory potency (Labiuk et al., 2019). These features make Thiorphan a promising candidate drug, warranting further research and development to explore its potential in treating related diseases.

#### 3.3.2 NEP, TLN, and ACE multi-target inhibitors

The three-dimensional model of thermolysin (TLN) has been widely used for identifying the active sites of NEP (Tiraboschi et al., 1999) and for inhibitor design (Ksander et al., 1997a). Previous research has identified R-Mercaptoacyl dipeptides such as compound Ala-Pro (Coric et al., 1996) and compound Gly-(5-Ph)Pro (Fournie-Zaluski et al., 1996), which bind to NEP through thiol-induced Zn2+ ion coordination and interactions with the enzyme’s1′ and S2′ subsites (Gaucher et al., 1999; Holmes and Matthews, 1981). Based on this discovery, they further validated the binding activity of three R-Mercaptoacyl dipeptides to NEP: [(2S)-2-sulfanyl-3-phenylpropanoyl]Gly-(5-Ph)Pro**26** (TLN IC_50_ = 1200 ± 200 nM; NEP IC_50_ = 1.6 ± 0.3 nM; ACE IC_50_ = 0.55 ± 0.05 nM, Figure 5D), [(2S)-2-sulfanyl-3-phenylpropanoyl]Phe-Tyr**27** (TLN IC_50_ = 42 ± 1 nM; NEP IC_50_ = 2.9 ± 0.6 nM; ACE IC_50_ = 5.6 ± 0.6 nM, Figure 5D), and [(2S, R)-2-sulfanylheptanoyl]-Phe-Ala**28** (TLN IC_50_ = 48 ± 5 nM, PDB ID: 1QF1; NEP IC_50_ = 2.9 ± 0.5 nM, Figure 5D) (Gaucher et al., 1999). Compounds **27** and **28** exhibit higher affinity than compound **26**. Comparing the inhibitory potency of these compounds and their various analogs against TLN, NEP, and ACE provides insights that confirm their binding modes with the latter two enzymes (Gaucher et al., 1999). A novel binding mode for thermolysin recognition, distinguished by the bidentate coordination of the zinc atom through thiol and oxygen groups, has been suggested. This concept opens up new avenues for designing inhibitors modeled after this interaction (Gaucher et al., 1999).

### 3.4 Clinical drugs

Entresto (LCZ696, sacubitril/valsartan) is an innovative pharmaceutical compound that integrates the functions of an ARB with the NEPI prodrug AHU 377, offering a dual-action approach for the treatment of heart failure and hypertension (Gu et al., 2010). LCZ696 is a novel ARNi that offers a dual therapeutic action. Upon oral ingestion, it swiftly initiates NEPI while simultaneously blocking angiotensin receptors, which is beneficial for managing heart failure and other cardiovascular conditions (Ksander et al., 1995). It is effective in treating hypertension and heart failure, showing antihypertensive effects in animal models and good tolerability in clinical trials with primarily mild adverse events (Ksander et al., 1995). In clinical studies, LCZ696 has demonstrated superior blood pressure reduction compared to placebo, AHU 377 alone, or valsartan alone in phase 1-2 hypertension trials (Ruilope et al., 2010). It has been demonstrated to elicit the anticipated alterations in plasma hormone levels, affirming the blockade of the AT1 receptor as evidenced by elevated plasma renin concentration, and the inhibition of neprilysin, as indicated by the increased concentrations of plasma ANP and cGMP (Ruilope et al., 2010).

The PARADIGM-HF clinical trial investigated the effects of sacubitril/valsartan, demonstrating that this medication significantly reduced the risk of cardiovascular death and heart failure hospitalization by 20% and the risk of all-cause mortality by 16% (Savarese et al., 2023). Regarding adverse reactions, the group treated with LCZ696 exhibited a reduced risk of experiencing cough, hyperkalemia, and renal dysfunction compared to the control group. Additionally, there were minimal treatment discontinuations due to symptomatic hypotension, and no significant differences were observed in the risk of angioedema between the groups (Savarese et al., 2023). Subgroup analyses highlighted LCZ696’s benefits in reducing sudden cardiac death, improving quality of life, enhancing metabolic profiles in type 2 diabetes patients, lowering uric acid levels, protecting renal function, and reducing heart failure rehospitalizations (Packer et al., 2018; Tomson et al., 2021; Jankowski et al., 2021; Ryu et al., 2021). Subgroup analyses highlighted LCZ696’s benefits in reducing sudden cardiac death, improving quality of life, enhancing metabolic profiles in type 2 diabetes patients, lowering uric acid levels, protecting renal function, and reducing heart failure rehospitalizations (Johansen et al., 2021). A study involving 3,518 HFrEF patients across 150 medical institutions recommended strengthening medication management and initiating LCZ696 therapy early and at adequate doses in eligible patients to improve clinical outcomes and prognosis (Johansen et al., 2021; Cai et al., 2013). Additionally, recent studies have shown that patients receiving a high dose of 400 mg of sacubitril/valsartan have a better prognosis compared to those on a low dose of less than 200 mg, with no significant differences in the incidence of hyperkalemia, serious renal events, and angioedema among the dosage groups (Doi et al., 2024). In another PARADIGM-HF trial, sacubitril/valsartan’s efficacy in reducing cardiovascular death or heart failure hospitalization was sustained or enhanced in patients with hypotension, highlighting its potential as a safe and effective treatment option in such cases (Matsumoto et al., 2024).

In a PARAGLIDE-HF trial’s secondary analysis, the win ratio (WR) was employed to evaluate the efficacy of sacubitril/valsartan compared to valsartan in heart failure patients with an ejection fraction >40% (Shoji et al., 2024). The primary hierarchical outcome, which included cardiovascular death, heart failure hospitalizations, urgent visits, and a 25% reduction in NT-proBNP, showed a significant treatment effect favoring sacubitril/valsartan (WR, 1.46; 95% CI, 1.08–1.97) in the subgroup with ejection fraction ≤60%. Sensitivity analyses, adjusting for NT-proBNP changes and incorporating renal outcomes, consistently supported the superiority of sacubitril/valsartan. These findings underscore the robustness of sacubitril/valsartan’s treatment benefit and suggest the value of pre-specifying WR sensitivity analyses in future studies for a comprehensive assessment of treatment effects. Another win ratio analysis of the PARAGON-HF study indicates that sacubitril-valsartan provides significant clinical benefits in patients with heart failure and preserved ejection fraction, with these benefits being independent of left ventricular ejection fraction and sex (Yoon et al., 2024).

## 4 Conclusion and perspectives

Neutral endopeptidase is a widely distributed membrane-bound zinc-dependent metallopeptidase that plays a critical role in various physiological processes, including diuresis, natriuresis, and the processing and degradation of vasoactive peptides. NEPIs are a class of drugs with significant clinical value, enhancing the physiological effects of bioactive peptides by inhibiting NEP activity. This makes them promising in treating Alzheimer’s disease, cardiovascular diseases, arthritis, and other conditions.

This review summarizes the research progress of NEPIs, focusing on single-target and dual-target inhibitors of NEP. Representative NEP single-target inhibitors such as Sacubitril and Thiorphan have demonstrated high efficacy and selectivity, marking them as promising candidates for further research. Current research on NEP dual-target inhibitors Mainly involves NEP with neprilysin and ACE and multi-target inhibitors, including ECE and DPP-IV. Despite their broad clinical potential, NEPIs' safety and side effects remain critical concerns in clinical application. NEPIs may cause hypotension, renal dysfunction, and electrolyte imbalances. Therefore, when using NEPIs, careful monitoring of physiological indicators and dosage adjustment according to individual patient conditions are essential. As research progresses, NEPIs' applications are expected to expand, and further investigation into their safety and side effects will help improve clinical outcomes.

The widespread distribution of NEP and its involvement in various crucial physiological processes make its inhibitors a vital research focus for treating multiple diseases. Although NEPIs offer many advantages, they also present challenges. For example, the binding affinity and stability of single-target inhibitors need further improvement, and there needs to be more comprehensive research regarding their efficacy and specificity. Future research may uncover new targets, leading to the design of more precise multi-target inhibitors. Additionally, personalized treatment plans and combination therapy strategies based on patients' genotypes and protein expression profiles could help optimize the use of NEPIs, enhance treatment efficacy, and reduce side effects.

In summary, NEP inhibitors hold significant potential for treating various diseases. Future research should explore their targets and combination therapies with other treatments to enhance their safety and broaden their application range, providing more effective and safer treatment options for patients.

## Glossary

NEP: neprilysin
NEPI: NEP inhibitor
NP: natriuretic peptides
Ang I: angiotensin I
Ang II: angiotensin II
Ang-(1-7): angiotensin-(1-7)
ACE2: angiotensin-converting enzyme 2
ADM: adrenomedullin
BK: bradykinin
NEPI: Neprilysin inhibitors
AD: Alzheimer’s disease
Aβ: β-amyloid
SAR: structure-activity relationships
ET-1: endothelin-1
GLP-1: glucagon-like peptide-1
DPP4: dipeptidyl peptidase-4
DN: Diabetic nephropathy
RAS: renin-angiotensin system
NPS: natriuretic peptide system
CKD: chronic kidney disease
ARNI: angiotensin receptor-neprilysin inhibitor
DR: Diabetic retinopathy
ARBs: angiotensin receptor blockers
SP: substance P
VIP: Vasoactive Intestinal Peptide
NPY: neuropeptide Y
HF: Heart failure
ACEI: ngiotensin-converting enzyme inhibitors
ARB: angiotensin II receptor blockers
RAAS: renin-angiotensin-aldosterone system
HFrEF: Heart failure with a reduced ejection fraction
ANF: atrial natriuretic factor
ANP: atrial natriuretic peptide
BNP: brain natriuretic peptide
